# Particulate matter in the workplace: effects of a mental models-based folder combined with a practical assignment

**DOI:** 10.1186/s12889-022-13362-y

**Published:** 2022-05-13

**Authors:** T. A. M. Stege, J. F. B. Bolte, L. Claassen, D. R. M. Timmermans

**Affiliations:** 1 National Institute for Public Health and the Environment (RIVM), PO Box 1, 3720 BA Bilthoven, The Netherlands; 2 Smart Sensor Systems Group, Faculty of Technology, Innovation, and Society, The Hague University of Applied Sciences, Rotterdamseweg 137, 2628 AL Delft, The Netherlands; 3 Department of Public and Occupational Health, Amsterdam Public Health Research Institute, Amsterdam University Medical Center, Amsterdam, The Netherlands

**Keywords:** Particulate matter, Work safety, Risk communication, Educational folder, Exposimeter

## Abstract

**Background:**

With increasing knowledge on the adverse health effects of certain constituents of PM (particulate matter), such as silica, metals, insoluble ions, and black carbon, PM has been under the attention of work safety experts. Previously, we investigated the perceptions of blue-collar workers in highly exposed areas of work. Subsequently, we developed an instruction folder highlighting the most important aspects of PM risk and mitigation, and tested this folder in a digital experiment. The digital experiment yielded positive results with regards to acquired knowledge about PM, but did not on risk perception or safety behavior.

**Methods:**

In this study, we investigate the effects of the folder when combined with a practical assignment involving a PM exposimeter, showing the amount of particulate matter in microgram per cubic meter in real time on its display for various activities. We tested this at six workplaces of four companies in the roadwork and construction branch.

**Results:**

The results indicate that the folder itself yields an increased knowledge base in employees about PM, but the effects of the practical assignment are more contentious. Nevertheless, there is an indication that using the assignment may lead to a higher threat appraisal among employees for high exposure activities.

**Conclusion:**

We recommend implementing our folder in companies with high PM exposure and focusing further research on appropriate methods of implementation.

**Supplementary Information:**

The online version contains supplementary material available at 10.1186/s12889-022-13362-y.

## Background

With increasing knowledge on the adverse health effects of certain kinds of PM (particulate matter), such as silica, iron and black carbon, PM has been under the attention of work safety experts. For the general public [1], but especially for people who work in environments with high PM exposure, such as the roadwork and construction branches [2–4], PM can be an important health risk. Adverse health effects include a wide array of respiratory and cardiovascular diseases [5], which are caused by a variety of different substances that make up the contents of PM, such as black carbon and metals [6, 7].

Although various means of protection against workplace PM exposure exist, not all employees that work in an environment with high PM exposure make sufficient use of these means of protection [8]. This discrepancy was the basis of a study on the situation in the roadwork and construction branches in the Netherlands, in which employees’ perceptions of risk and mitigation were identified [9], with the intention of empowering workers in these branches to work more safely in an environment with high PM exposure. We did this based on a mental models approach, in which discrepancies between blue-collar workers’ views on PM and experts’ views on PM are the basis of which information to include in the communication of risks and mitigation [10–12].

In our earlier studies [13, 14], we developed and tested a folder about workplace PM exposure and its mitigation. The folder turned out to improve participants’ knowledge about PM in a digital experiment [14]. However, effects on risk perception variables and safety behavior, as seen in the Protection Motivation Theory (PMT) [15], could not be demonstrated. Although the effects on PM knowledge in the digital experiment were promising, the extrapolation to potential effects in the workplace is nontrivial. This is partly because the population composition of the digital experiment may be slightly different from that of an actual company, but also because the instructions in work safety meetings may be given in a different way than in an online experiment.

The absence of the opportunity for active learning in the digital experiment may explain the lack of effect on risk perception and safety behavior. Based on the SECTIONS model [16], we investigated the optimal means of instruction. A practical assignment that visualizes the exposure in the working environment with and without mitigation methods in place may induce active learning [17]. An exposure visualization assignment like this has been used in a similar setting with promising results [18]. We did not test such an assignment in the digital experiment, as a personalized workplace setting could not feasibly be simulated.

The present field study on PM risk communication in the workplace is aimed at answering two main research questions. Firstly: ‘To what extent does an intervention involving our educational material lead to more knowledge and a better risk perception about PM when used in a workplace setting?’ Secondly: ‘Is the intervention involving our material more effective if it is augmented with a practical assignment?’ Based on our earlier experiences, we expect our educational folder to at least improve employees’ knowledge about PM, In addition, based on the potential positive effect on active learning we expect that the practical assignment will affect threat and coping appraisal.

## Methods

### Participants

We contacted various companies in the roadwork and construction branches, i.e. branches whose employees tend to have high PM exposure, from our professional network in the Netherlands. Four of those companies agreed to participate in our study. The four companies found 74 participants able and willing to participate in total; however, of these 74, 17 dropped out during the study or failed to adequately fill in the study questionnaires. The four companies respectively had 24, 8, 16 and 9 employees completing the experiment successfully, yielding 57 participants in total. Of these 57 participants, 55 were male, which appears to be representative of the roadwork and construction branches as a whole, as we found similar male-dominated demographics in earlier studies [9]. Participants’ ages ranged between 17 and 65, with an average age of 42.9.

### Design

This study can be seen as a mixed methods study, albeit with a larger focus on the quantitative part. For the quantitative part, all participants were subjected to a pretest and a posttest. These were two identical questionnaires, meant to investigate the differences in PM knowledge and risk perception before and after the intervention. The intervention itself consisted of two parts. The first part was a work safety meeting, in which participants were each given the educational folder about PM that we developed and tested in earlier studies [13]. An instructor (either the work safety specialist or the first author of this article) summarized the key points of this folder, provided some additional information, and answered questions. The second part was a practical exposure visualization assignment.

We use a step-wedge design, in which some participants are given the practical assignment before the final questionnaire, and some after. The latter is not strictly necessary to investigate the effectiveness of this practical assignment, but it could prove useful for the companies themselves even beyond the scope of this study. Furthermore, we felt that all participants should at least get the opportunity to work with the PM dosimeters. In this way, we managed to make a clear distinction between conditions with and without the practical assignment on the posttest, without withholding a potentially valuable learning experience from half of the participants.

For the smaller qualitative part of this study, we compiled a logbook of each of the instances of data collection. For these four companies, there were six instances of data collection in total, as two companies had two locations each. Any noteworthy questions, complications and important moments from each of these six instances were written down in the logbook. Furthermore, work safety experts of all four companies involved in this study, who were all present during the intervention, were asked to answer a few questions by email after participating in our study.

### Materials

The questionnaires involved five multiple choice questions related to PM knowledge (related to properties of PM, its causes, its effects, and the mitigation methods), the answer to which could be found in our folder. It also involved seven questions related to risk perception; three of these seven questions are related to the PMT variable of ‘threat appraisal’, three to ‘coping appraisal’, and the final question is about safety behavior, i.e. to what extent people work safely in practice with regards to PM. These seven questions are answered with five-point Likert scales. The full questionnaire can be found in Additional file 1: Appendix A. All questions, both the knowledge and the perception questions, were used earlier in the larger online questionnaire from our previous study [14]; we made a smaller selection of the most relevant questions in order to decrease workload for participants, as they should not be held from their job longer than strictly necessary.

The longer questionnaire from our previous study [14] was validated in the following way. For the PMT-related questions, we used questions that were very similar to those in existing PMT research, and we performed reliability analyses afterwards to confirm the internal consistency of the measures constructs. For the knowledge questions, we discussed the answers with experts in the field of PM and risk communication. We did not perform any additional checks for this smaller questionnaire, but it is unlikely that any new problems should arise related to validity or reliability.

**Fig. 1:**
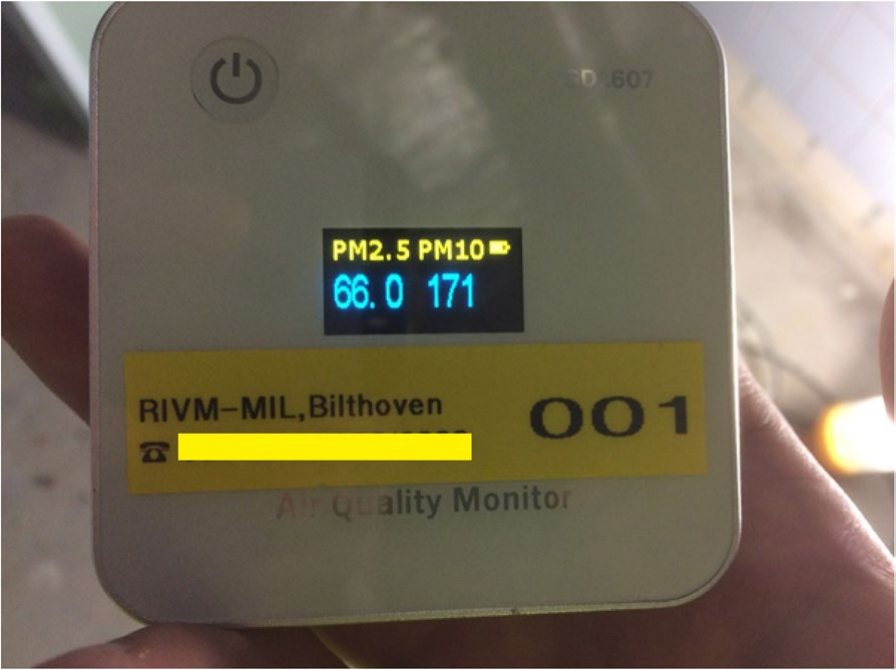
A low cost, portable PM exposimeter with real time display of the amount of microgram per cubic meter, Nova Fitness SDL607. (Phone number censored)

The small follow-up email questionnaire for the involved safety experts contained eight open questions such as ‘To what extent do you think employees learned something from the practical assignment?’ The full questionnaire can be found in Additional file 1: Appendix B. Finally, for the practical assignment, portable PM exposimeters are given to participants, and they are prompted to answer questions about PM exposure in their company. The full assignment can be seen in Additional file 1: Appendix C. A photograph of the exposimeters used in this study can be found in Fig. 1.

### Procedure

The data was collected during the year 2020, in which some companies had to postpone participation due to COVID-19 restrictions. The remaining companies were visited in the summer, and all participants, work safety specialists and researchers made sure to comply with COVID-19 related regulations. All four participating companies were visited on weekdays, during a timeframe in which they were already planning to organize a periodical work safety meeting. These meetings are required by law in the Netherlands for roadwork and construction companies.

Participants were gathered in a room (sufficiently large enough to maintain COVID-19 regulations if applicable), and as the meeting started, they were asked to fill in the pretest questionnaire. These questionnaires were clearly marked to avoid confusion with posttest questionnaires (as they are otherwise identical). After filling in the pretest questionnaire, the participants received a physical copy of the folder, and they were prompted to read it. The work safety specialist was then asked to tell something about PM based on the contents of the folder. Then, upon compliance of the work safety specialist, the group was split in half, as randomly as possible. One half of the group (the ‘safety meeting only’ group)^1^ received the posttest questionnaire immediately, and the other half (the ‘added assignment’ group) was given the practical assignment as well as a PM meter. After both groups were finished, they switched. We made sure that as many participants as possible filled in both the pretest and posttest questionnaires, and we answered questions during the assignment if needed. We wrote down the most important observations during this assignment in our logbook. After finishing the work safety meeting, we emailed the work safety specialist the short qualitative questionnaire.

### Analyses

For the quantitative analysis, we compiled a dataset based on the pretest and posttest questionnaires in SPSS version 16. First, we checked whether there were any differences in demographic variables between conditions and companies, such as gender, level of education (both by chi-square tests), age and work experience (both by ANOVAs). We recoded the answers to the different knowledge-related questions into ‘correct’ (1) or ‘incorrect’ (0), and subsequently compiled a knowledge sum score.

We analyzed the reliability of the scales of threat appraisal and coping appraisal in order to check whether the questions could indeed be viewed as part of a larger scale and calculated average scores (for threat appraisal and coping appraisal). The reliability analysis revealed that the threat appraisal scale has a Cronbach’s α of 0.83, which is sufficient for use as a coherent scale. We therefore computed threat appraisal scores on the pretest and posttest by calculating an average of the scores on each of the three items. A coherent scale could not be constructed for coping appraisal. The Cronbach’s α with all four items included was 0.31; inter-item correlations ranged from smaller than 0.01 to 0.41; and removing any item from the scale could not increase Cronbach’s α any further than 0.46. Therefore, we decided to view the coping appraisal items not as a scale, but as independent items.

For each of the test scales, we analyzed the differences between the pretest and posttest scores using a paired-sample t-test, to investigate any significant general effects of the intervention. In addition, we performed univariate ANOVA’s of condition (‘safety meeting only’ versus ‘added assignment’) and company on the three posttest scores, corrected for the respective pretest scores and potential differences in demographic characteristics between conditions and companies.

We also performed a general analysis of the findings from the logbook and the qualitative questionnaire for the work safety experts. The amount of information from these sources was not sufficient to perform any detailed qualitative analyses. We selected the information specifically relevant for the interpretation of the quantitative information.

## Results

### Demographics

We performed a series of checks to investigate any bias between various groups when it comes to demographics. For the two conditions – the ‘safety meeting only’ and the ‘added assignment’ condition – we performed chi-square tests with gender and level of education. The results of the chi-square tests revealed no significant differences between the two conditions regarding gender (χ^2^ = 0.03, df = 1, *p* = 0.86), nor regarding level of education (χ^2^ = 3.69, df = 3, *p* = 0.30). Then, the four companies involved were checked for the same biases, with two additional chi-square tests with gender and level of education. The results of the chi-square tests revealed no significant difference among the four companies regarding gender (χ^2^ = 3.47, df = 3, *p* = 0.33). However, a significant difference was found among the four companies regarding level of education (χ^2^ = 25.26, df = 9, *p* < 0.01), meaning that not all companies provided similarly educated participants. In company 3, there were significantly more educated participants than in company 1 or 4 (25% or 4 out of 16). Company 2 also had 25% of their participants higher educated, but also 50% of their participants with a lower or no additional education (beyond grade school).

To check any biases between companies and conditions regarding work experience or age, a series of one-way ANOVAs was conducted. These ANOVAs revealed no significant differences between companies regarding age (F = 0.56, df = 3, *p* = 0.65) or experience (F = 0.77, df = 3, *p* = 0.51), nor were there any significant differences between the two conditions found regarding age (F = 0.18, df = 1, *p* = 0.67) or experience (F = 0.38, df = 1, *p* = 0.54).

### Knowledge scores

To acquire a general idea of the difficulty of the questions, as well as the effects of the work safety meeting, we first calculated the percentages of correct answers for each of these knowledge questions. The results can be found in Table 1. As can be seen from the table, the number of correct answers appeared to increase with each question, except for the question about weather effects on PM; almost all participants already knew the answer to that question before the meeting.

**Table 1:** Percentages of correct answers on the knowledge questions

Question #	Topic	Percentage correct (pretest)	Percentage correct (posttest)
1	Visibility of PM	61%	72%
2	Diseases caused by PM	16%	35%
3	Weather effects on PM	98%	95%
4	Mitigation of PM	88%	90%
5	Diesel as a source of PM	33%	44%

**Fig. 2:**
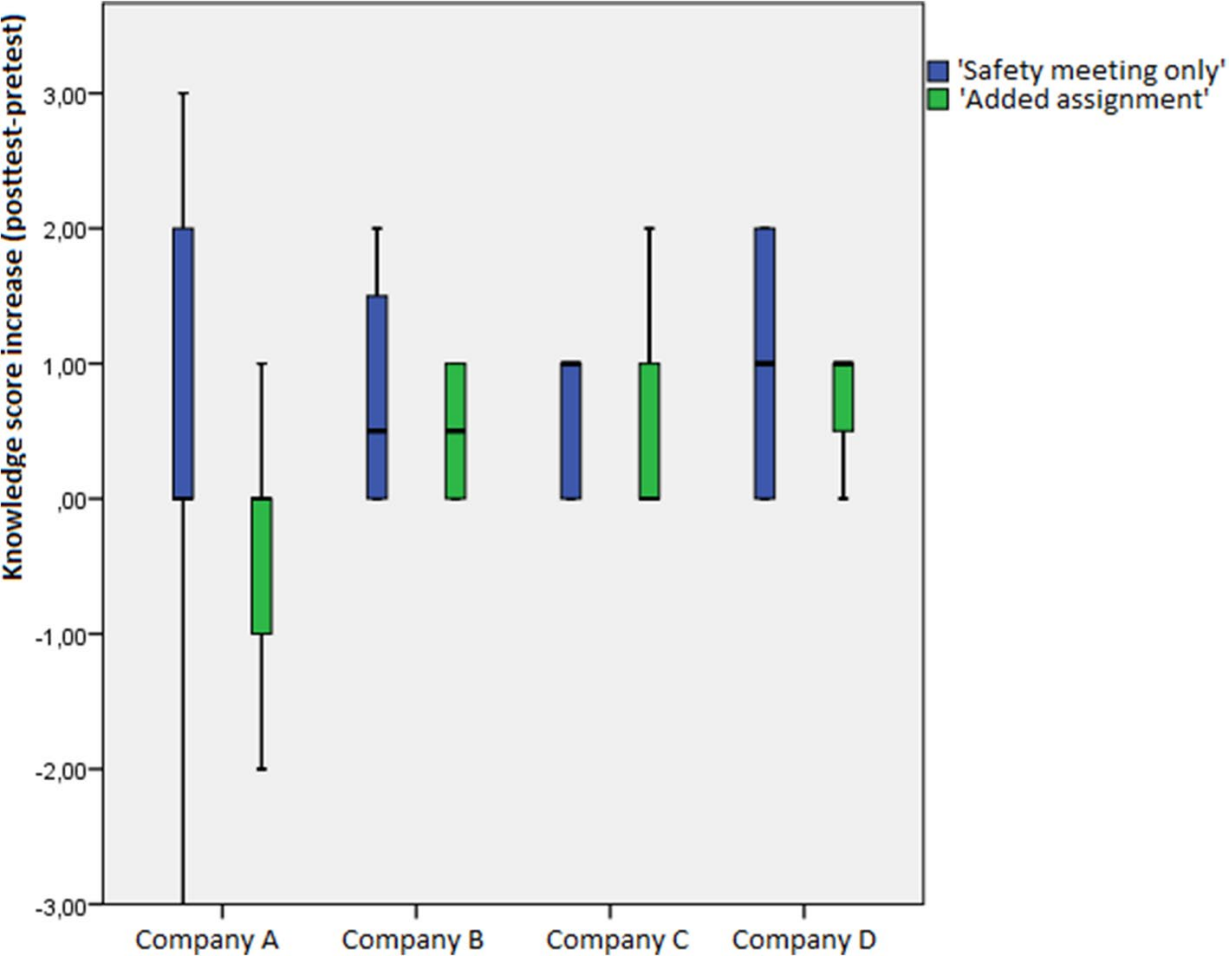
Knowledge score increases for all employees in each of the companies, split by condition

**Fig. 3:**
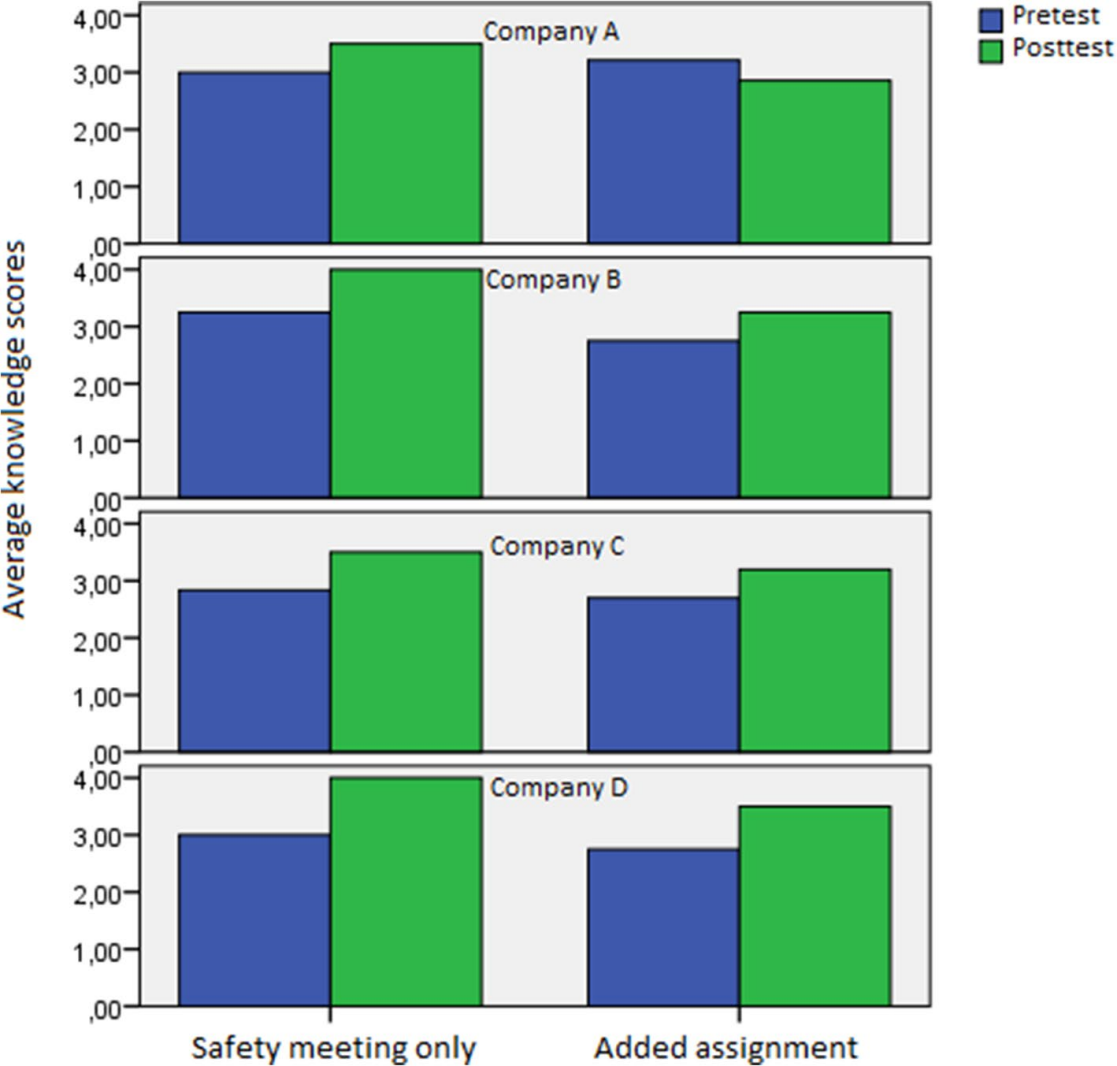
Average knowledge scores on the pretest and posttest, split by company and condition

The average knowledge score (on a scale from 0—5) increased from 2.96 before the meeting to 3.35 after the meeting (t = -2.88, df = 56, *p* < 0.01). It is noteworthy that, even though many participants show an increase in score (23 in total), there are a few participants (6 in total) who show a decrease in score, one of whom even has a decrease of 3 points; all of these 6 participants with a decrease in score are found in Company A (two in one workplace of Company A and four in the other), and 5 of these 6 participants are found in the ‘added assignment’ condition within Company A. This is further shown in a boxplot in Fig. 2. The other 28 participants had no score change at all. A more detailed overview of average scores in each of the companies, split by condition, can be found in Fig. 3.

We found that the posttest scores, corrected for pretest scores and level of education, were significantly influenced by condition (F = 10.89, df = 1, *p* < 0.01); people in the ‘safety meeting only’ condition performed significantly better than people in the ‘added assignment’ condition. The effect of the company was not significant (F = 2.02, df = 3, *p* = 0.13). No significant interaction effects of company and condition were found either. However, since all of the 6 participants with a decrease in score are found in Company A, and the process of the work safety meeting had some variations in Company A when compared to the other companies (we will discuss this in more detail in the process evaluation portion of this article), we performed a follow-up analysis without Company A. Nevertheless, people in the ‘safety meeting only’ condition still performed significantly better than people in the ‘added assignment’ condition, when considering only companies B, C, and D (F = 8.33, df = 1, *p* = 0.01). The effect of the company now becomes significant as well (F = 3.76, df = 2, *p* = 0.048), with participants in Company C performing significantly worse than the other companies. There are still no significant interaction effects.

### Threat appraisal

**Fig. 4:**
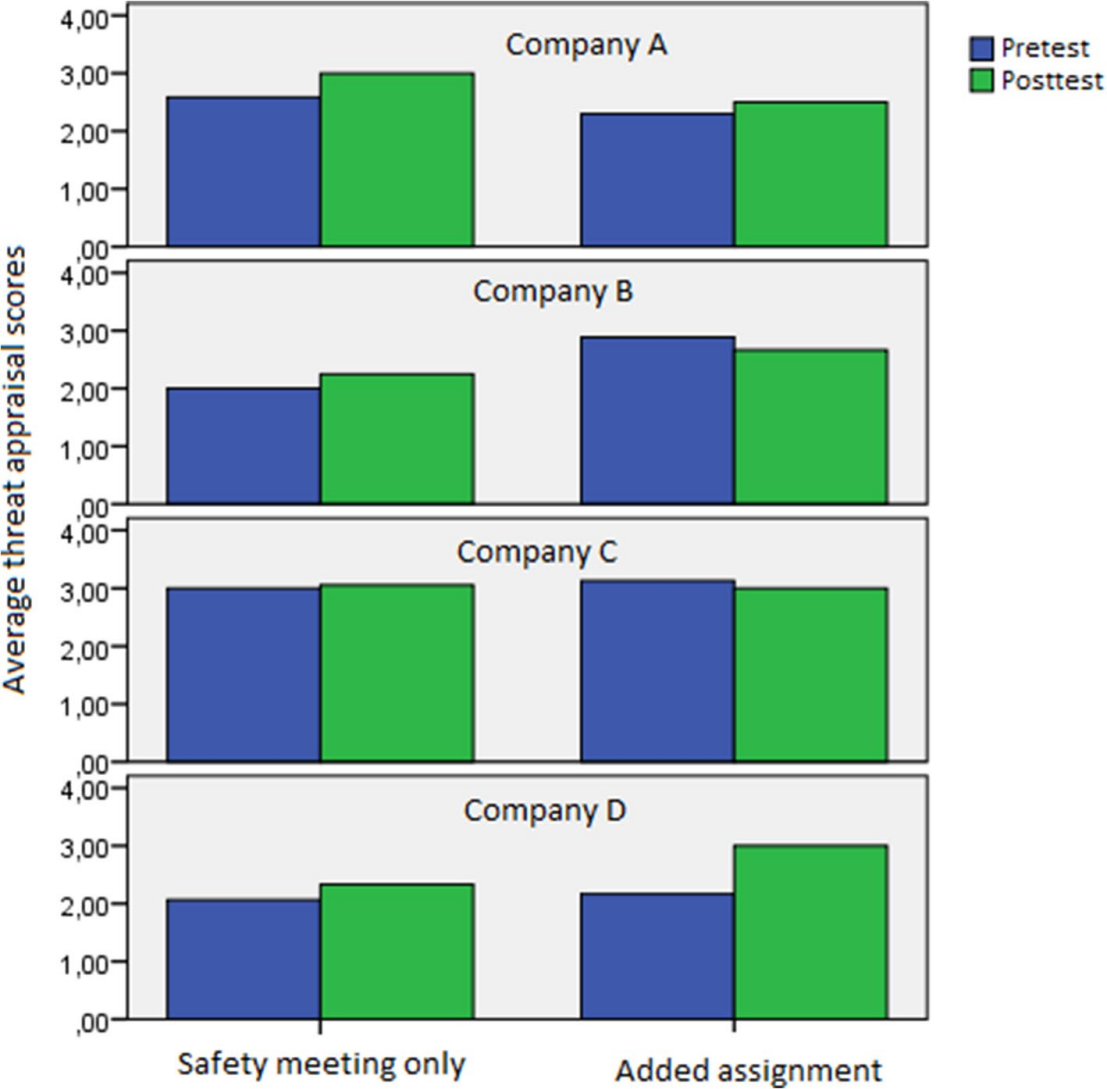
Threat appraisal scores on the pretest and posttest, split by company and condition

We compared the threat appraisal scores of the pretest and posttest. Overall, an increase in threat appraisal was found, with threat appraisal scores on average increasing from 2.55 to 2.74. A paired-samples t-test revealed that this increase was significant (t = -2.11, df = 53, *p* = 0.04). An overview of all scores among different companies and conditions can be found in Fig. 4. We performed another univariate ANOVA, investigating the effects of company and condition on posttest threat appraisal scores, corrected for pretest threat appraisal scores and level of education. We found no significant effects of condition (F = 0.61, df = 1, *p* = 0.44) or company (F = 1.28, df = 3, *p* = 0.30). We also found no significant interaction effects. A follow-up analysis without Company A did not give any significant effects either.

### Coping appraisal

The paired-sample t-tests on each of the four coping appraisal items showed that only one of these four items, which involved the statement *‘I know how to protect myself against particulate matter’*, revealed a significant increase: average scores on this item increased from 2.56 on the pretest to 2.91 on the posttest (t = -2.47, df = 53, *p* = 0.02). No significant effects were found for the other three items.

**Fig. 5:**
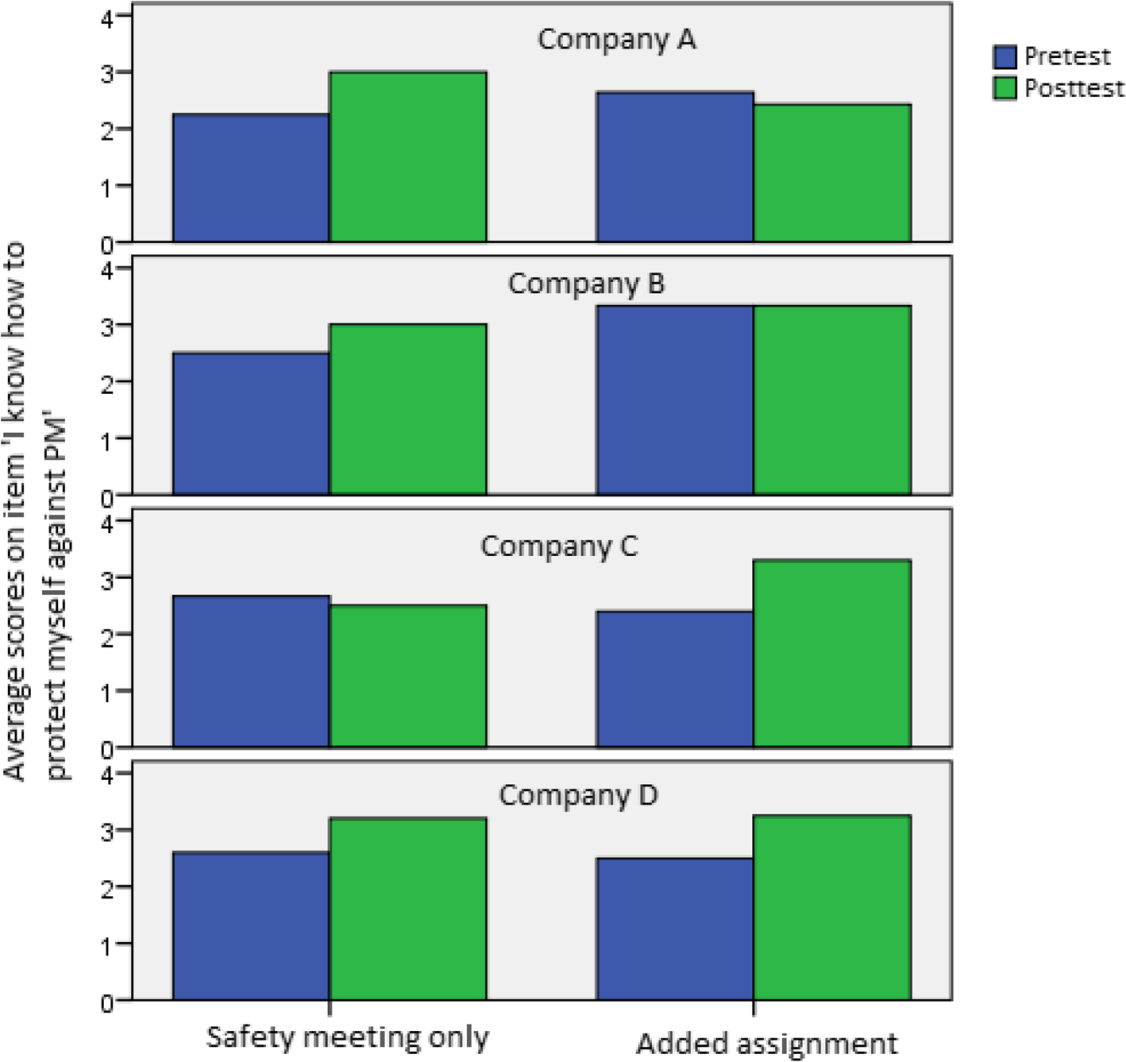
Scores on the item ‘I know how to protect myself against PM’ on the pretest and posttest, split by company and condition

The univariate ANOVA on the posttest score of just this item of the coping appraisal scale, correcting for the pretest score of the same item and level of education, showed no significant direct effects of condition (F = 1.75, df = 1, *p* = 0.20) or company (F = 0.35, df = 3, *p* = 0.79). However, we did find a significant interaction effect of company and condition (F = 5.10, df = 3, *p* < 0.01). Figure 5 showcases all average scores on this item among various companies and conditions.

We again performed a follow-up analysis without Company A. In this case, the interaction effect of company and condition disappeared (F = 2.51, df = 2, *p* = 0.12). No other significant effects were found either.

### Process evaluation: logbook

The first practical issue was related to making sure that the same participants filled in both the pretest and posttest questionnaires. Originally, we planned to pre-emptively send the pretest questionnaire to the work safety experts who coordinated the meetings within the companies, so the participants could fill them in before the meeting. This would reduce the amount of time lost on questionnaires during the work safety meetings, as well as reduce potential annoyance by participants of filling in the same questionnaire twice over a short time. However, upon visiting company A, we found that this plan had the added downside of causing a relatively large dropout rate in company A (8 out of 32 potential participants). Also, not all participants filled out the forms before attending the safety meeting, leading to irritation and a slow start of the safety meeting as some participants had to wait while others were filling out their forms. For this reason, we decided to give the pretest questionnaires to the other three companies during the work safety meetings.

Another practical issue that should be noted was related to the person presenting the work safety meeting. To fully test the usability of the folder by these companies, we had planned the work safety meetings to be carried out by the experts who are employed by these companies. In company A, this is indeed what happened; the work safety expert presented the information in the folder as the participants listened and had the opportunity to read the folder in the meantime. In company B, however, the work safety expert did not feel confident enough about presenting the information in the folder and instead felt that this task would be more suitable for the first author of this article. As this had the additional benefit of more thoroughly focusing on the most relevant subjects, we decided to maintain this practice for the final two companies.

When it comes to the practical assignment with the PM meters, there are large differences not just between companies, but also between locations within a single company. Some participants managed to find very high PM exposures of more than 999 µg/m^3^, the maximum value the PM meter could show, which is several orders of magnitude higher than recommended. This value of 999 µg/m^3^ was found during a wide array of activities, ranging from using heavy machinery and drilling equipment to cleaning the floor with a broom or with compressed air. However, in other companies or locations, PM exposures were relatively low, especially when working outside. One of the companies (B) was visited on a cold and rainy day with a below-average amount of traffic, resulting in low PM exposures for the workers outside, consistently below 20 µg/m^3^.

### Process evaluation: work safety expert opinions

Overall, the four work safety experts that were connected to the four companies involved with this study were all satisfied with the procedure, and gave positive feedback on the folder as well as the practical assignment. Work safety experts’ opinion about the folder is illustrated by the following quote:

*“The folder is compact and assembled in understandable language for my colleagues. They are usually not so keen on scientific language.” (Expert Company A)*

The work safety experts also agree on the added value of the practical assignment with the PM meter:

*“A work safety meeting should preferably be interactive.” (Expert Company A)*

*“Performing the measurements was great, people are still talking about it today. […] They were skeptical at first, but after performing the measurements, this was over.” (Expert Company B)*

*“Due to the measurements, people have become more aware about what PM is.” (Expert Company D)*

Some experts gave a couple of suggestions for improving the work safety meeting as a whole:

*“A fitting short movie as an introduction would make it more complete. […] In the end, talking through the results of the measurements.” (Expert Company B)*

One expert did point out that a briefing for the work safety experts would be useful (although three of the four experts did not find this necessary):

*“A short explanation by means of an instruction movie. This should at least involve […] information about the subject, […] the goal of the work safety meeting, [… and] how to give the work safety meeting.” (Expert Company C).*

## Discussion

In this study, we investigated the effects of a mental-model based instruction folder about PM for use in the workplace. We assessed whether using the folder led to an increase in knowledge and risk perception about PM, and whether augmenting the intervention with a practical exposure visualization assignment led to any improvements in effectiveness. We will argue that, while not all of the expected effects that we measured were significant, the results of this study are still promising when it comes to the effectiveness of our intervention.

### Knowledge scores

Based on the increase in knowledge scores, the folder can be shown to succeed in giving the participants basic information about PM, regarding its causes, effects, properties and methods of mitigation. This finding corroborates our earlier findings in a digital experiment [14], that also showed an increase in PM knowledge. As the present study focuses more on the actual end user of the folder, these results are potentially even more relevant.

People who were given the added assignment before the posttest scored significantly *lower* on the knowledge scores than people who were given the posttest directly after the safety meeting. This was contrary to expectations, but it may be explained by the effect of time; the participants who were busy performing the assignment had more time to forget the exact information needed to answer the questions correctly. Furthermore, they may also have been less conscientious when filling in the questionnaire because they had been busy with the assignment for quite a long time, which may have caused them to lose concentration.

If PM knowledge deteriorates quickly, this may become a problem when giving information is one of the main goals. This problem, however, may be related to the nature of the questions; perhaps we should not expect workers in a practical setting to have exact long-term knowledge on the subject, but we should focus on awareness instead. Judging by the comments of the experts on the practical exposure visualization assignment, these experts at least perceive an increase in long-term awareness about PM, which they perceive to be predominantly caused by the addition of the practical assignment.

Company A is the only company in which some participants show a *decrease* in PM knowledge after the work safety meeting. The explanation may be found in two of the anomalies we described in our logbook. Company A was the only company in which participants were given the pretest questionnaire a few days before the work safety meeting, and also the only company in which not the researcher, but the institutionalized work safety expert gave the PM-related information during the meeting. It is possible that the participants had forgotten which answers they gave during the pretest, since it was a few days before the meeting, and subsequently became confused about the information given by the work safety expert, since there may be small anomalies between the framing of the information by the expert and the information in the folder. This potential problem exemplifies the recommendation given by one of the experts in another company, to include a briefing for the person giving the work safety meeting. It should be noted that we followed the protocol as closely as possible in each of the companies beyond these two differences, so the situations between companies should still be sufficiently comparable.

### Threat and coping appraisal

Contrary to our previous study in an online setting [14], we did find a small, but significant increase in both threat and coping appraisals after this work safety meeting. Regarding threat appraisal, it did not appear to matter much whether or not the assignment was added, nor in which company they were active; on average, participants felt slightly more aware of the potential risks of PM after receiving the toolbox. The absence of differences between companies and conditions means that differences between measured exposure – which were noteworthy, as mentioned in the process evaluation – do not appear to translate to differences in threat appraisal.

With coping appraisal, however, it appears that performing practical exposure visualization assignment increased the workers confidence in how to protect themselves against particulate matter, except for the workers in company A, where it lead to a decrease in confidence l. The exact cause of this discrepancy is unclear, but as mentioned before, company A did have a somewhat different procedure of carrying out the work safety meeting and the questionnaires than the other companies. It is possible that a more consistent procedure would have led to more consistent results.

### Strengths and weaknesses

Overall, it appears that the main strength of this study is its strong alignment with what a work safety meeting might look like in practice. The findings from an earlier digital quantitative survey were corroborated and, to some extent, even expanded on by involving the actual end user of the intervention. The researchers made every effort possible to ensure that the work safety meetings would follow the guidelines of the various companies that participated, thereby increasing the generalizability of the results of the study.

Nevertheless, the study also has some weaknesses. As mentioned before, the inconsistency of the procedure may have harmed the consistency of the results, especially since the deviant company was also the one with the most participants. Adding more companies would not have been a feasible solution if we wanted to maintain consistency in the type of companies involved; adding companies from another branch than simply roadwork and construction could have been useful for increasing the number of participants, but may also have decreased the reliability of the results. For this reason, we decided to maintain the current selection of companies despite the issues with the procedure.

Within the companies, there may have been some selection bias when it comes to the participants in this study. For example, participants who are highly disinterested in PM may fail to submit a posttest questionnaire, thereby skewing the results in favor of those participants who are interested in learning more about PM as an occupational exposure risk. Nevertheless, in general, the participants who submitted pre- and posttest questionnaires appeared to form a representative sample of the company they were a part of.

## Conclusion

The mental models-based instruction folder has been shown to have a positive effect on workers’ knowledge about PM. This was shown both in an earlier digital survey and in this current practical study. There is a noticeable increase in participants’ knowledge about PM after taking part in a work safety meeting based on the contents of our instruction folder.

The effects of the added assignment appear to be more contentious at present. The expected positive effects on threat and coping appraisal are still somewhat dubious, and the assignment may even have a negative effect on the direct retention of knowledge. Nevertheless, the fact that three of the four companies do show an increase in coping appraisal for the participants who did the assignment before the final questionnaire does make the added assignment promising as a means of increasing the work safety.

All in all, the mental models-based folder has proven to be a useful tool for use in occupational work safety meetings. We recommend looking into the option of including a briefing for the work safety experts, to decrease potential procedural difficulties. This may be a topic for future research. Mainly because of the enthusiasm within the companies, a practical assignment similar to ours may still be a useful addition as well; however, the set-up of the measurement assignment (duration, potency for exposure contrasts at the workplace) could be redesigned. Finally, one of the experts recommended adding a small instruction movie, which is also something that could be investigated.

## Supplementary Information

**Additional file 1: Appendix A.** Questionnaire for the workers (translated from Dutch). **Appendix B.** Open email questionnaire for the work safety experts. **Appendix C.** Practical assignment with the PM exposimeter.

## Data Availability

The datasets used and/or analysed during the current study available from the corresponding author on reasonable request.

## Abbreviations

ANOVA: Analysis of variance
COVID-19: Coronavirus Disease 2019
PM: Particulate Matter
PMT: Protection Motivation Theory
RIVM: Rijksinstituut voor Volksgezondheid en Milieu (National Institute for Public Health and the Environment)
SECTIONS: Students, Ease of use, Costs, Teaching, Interactivity, Organizational issues, Novelty and Speed
SPR: Strategic Programme RIVM
UMC: University Medical Center
WHO: World Health Organization
WMO: Wet Medisch wetenschappelijk onderzoek met mensen (Medical Research Involving Human Subjects Act)

## Footnotes

1 It should be noted that, when naming the two conditions in this study, we define the ‘safety meeting’ as the combined intervention of the folder with the oral instructions, but without the assignment. Therefore, the ‘safety meeting only’ condition did receive the folder before the posttest questionnaire, but not the assignment.
