# Supplementary material for: Particulate matter in the workplace: effects of a mental models-based folder combined with a practical assignment
Source: BMC Public Health. 2022 May 13;22:968. doi: 10.1186/s12889-022-13362-y (PMC9106267; doi:10.1186/s12889-022-13362-y)
Supplement: Supplementary file 1 — Additional file 1: Appendix A. Questionnaire for the workers (translated from Dutch). Appendix B. Open email questionnaire for the work safety experts. Appendix C. Practical assignment with the PM exposimeter. [file 12889_2022_13362_MOESM1_ESM.doc]

## Appendix A. Questionnaire for the workers (translated from Dutch)

*Note: this part was detached from the rest of the questionnaire to facilitate anonymization*

This questionnaire is part of a study by the RIVM and Amsterdam UMC about particulate matter in the workplace. We would like to get an impression of your ideas about particulate matter (PM). We will also ask you some questions about your knowledge of PM. Please try to answer these questions as accurately as possible. If you are unsure about an answer, please fill in what you think might be correct. Filling in this questionnaire will take about 10 minutes.

Your personal answers or data will not be given to third parties and will be treated confidentially. You will have the opportunity to view your own data. You are always free to participate or to stop participating whenever you want. If you choose to stop participating, already given answers will be deleted.

Do you understand this text and do you agree with these terms? YES / NO

Name: _______________________________________________

Respondent #: _______________________________________________

Thank you for participating!

---

**General question**

- Did you get any information about PM in the workplace in the last 2 years? YES / NO

**Knowledge questions**

We will now ask you ten knowledge questions about particulate matter (PM). Please try to answer them as accurately as possible. Please circle the answer you think might be correct, even if you are unsure.

1. Which statement about PM is true?
   1. PM is always visible.
   2. PM is usually visible, except in very low concentrations.
   3. *PM is usually invisible, except in very high concentrations.*
   4. PM is never visible.
2. Which of the following diseases **cannot** be caused by PM (as far as is known)?
   1. Stroke
   2. *Colon cancer*
   3. Heart failure
   4. Lung cancer
3. Which kind of weather increases PM risk?
   1. *Drought.*
   2. Rainy weather.
   3. Storm.
   4. Extreme cold.
4. What is the best way to mitigate PM exposure when sawing or drilling?
   1. Treating the material in a closed-off space.
   2. Treating the material with outstretched arms.
   3. *Wetting the material before use.*
   4. Treating the material as quickly as possible.
5. Which is the following statements is true?
   1. Ventilation systems often do not work against PM.
   2. *Diesel causes more PM exposure than gasoline.*
   3. To prevent PM exposure, people should only work at night.
   4. Employees are fully responsible for their own protection against PM.

**Opinions on particulate matter**

These questions will ask you about your personal views on particulate matter (PM). For each of the following statements, please answer to what extent you agree with it, on a scale from 1 (completely disagree) to 5 (completely agree).

- I think that the health risk of PM in my workplace is large.

- - Completely disagree / Disagree / Moderately agree / Agree / Completely agree
- Exposure to PM could make me severely ill.
  - Completely disagree / Disagree / Moderately agree / Agree / Completely agree
- I am worried about PM exposure at work.
  - Completely disagree / Disagree / Moderately agree / Agree / Completely agree
- I know how to protect myself against PM.
  - Completely disagree / Disagree / Moderately agree / Agree / Completely agree
- I think it is inconvenient to protect myself against PM.
  - Completely disagree / Disagree / Moderately agree / Agree / Completely agree
- I think it makes sense to protect myself against PM.
  - Completely disagree / Disagree / Moderately agree / Agree / Completely agree
- When performing work that gives PM exposure, I use protection against PM.
  - Completely disagree / Disagree / Moderately agree / Agree / Completely agree

**Personalia**

- Respondent #: ________________________________________________
- Age: _____________________
- Gender: male / female / other
- Occupation: _______________________________________________
- Years of work experience (current profession): ______________________
- Highest complete education: _________________________________________

(C) / (E)

## Appendix B. Open email questionnaire for the work safety experts

- How did you experience giving this work safety meeting with our folder?
- To what extent do you feel the work safety meeting improves knowledge and attitudes towards PM?
- To what extent did the practical assignment with the PM exposimeter form a worthwhile addition?
- What do you consider the best method of giving the work safety meeting?
- How involved do you think workers feel when you give a work safety meeting such as this, and how would you try to increase involvement?
- Is a work safety meeting like this compatible with your company culture, and why?
- To what extent are the mitigation methods against PM that are mentioned in the folder relevant and attainable?
- How would you like to get any instructions, if any, for giving a work safety meeting about PM?

## Appendix C. Practical assignment with the PM exposimeter.

You will be given a PM exposimeter for this assignment. The screen shows two numbers, you should look at the number on the right side of the screen (the higher number), denoting the total exposure to PM. Please answer the following questions about the PM exposure:

| How high was the exposure at the office? | ______________________ microgram/m^3^ |
| --- | --- |
| How high was the exposure in the workshop / practical workplace? | ______________________ microgram/m^3^ |
| Which work situation gave the highest PM exposure you could find? How high was this exposure? | ______________________ microgram/m^3^  Situation:  ____________________________________ |
| Which work situation gave the lowest PM exposure you could find? How high was this exposure? | ______________________ microgram/m^3^  Situation:  ____________________________________ |
| How often do you think you are exposed to the highest exposure level you could find? | _________________________ |
| How high would you think your average exposure at work would be? | ______________________ microgram/m^3^ |
